# Supplementary material for: IL-17C is a driver of damaging inflammation during Neisseria gonorrhoeae infection of human Fallopian tube
Source: Nat Commun. 2024 May 4;15:3756. doi: 10.1038/s41467-024-48141-3 (PMC11069574; doi:10.1038/s41467-024-48141-3)
Supplement: Supplementary file 1 — Supplemetary Information [file 41467_2024_48141_MOESM1_ESM.pdf]

# Supplementary Information

## Supplementary Methods

### Mass spectrometry analysis of cell-free supernatant

*Sample preparation*

To assess membrane-associated proteins in the cell-free supernatant, filtered supernatant (n=1) was ultracentrifuged for 1 hr at 400,000 x g using a TLA-110 rotor. Pelleted outer membrane vesicles were washed and resuspended in 20µl water. Subsequently diluted with 80µl of 1% SDS, 2mM EDTA and 20mM Tris pH 7.5 then sonicated for 3 minutes at room temperature in bath sonicator. Proteins were precipitated with addition of TCA (10% final) and acetone (50% final), washed in cold acetone and finally resuspended and denatured in 30 µl of 8M urea. 200 µl total volume trypsin/LysC digestion was carried out next where the samples were first diluted to 120µl final volume with 5µl of 25mM DTT and 85µl 25mM NH4HCO3 (pH8.5) for the reduction step, which was carried out for 15 minutes at 56°C. After cooling on ice to room temperature 6µl of 55mM CAA (chloroacetamide) was added for alkylation where samples were incubated in darkness at room temperature for 15 minutes. This reaction was quenched by 16µl addition of 25mM DTT. Subsequently 10µl of Trypsin/LysC solution [100ng/µl 1:1 Trypsin (Promega):LysC (FujiFilm) mix in 25mM NH4HCO3] along with 48µl of 25mM NH4HCO3 (pH8.5) was added to the samples for a final 200µl volume. Digests were carried out overnight at 37°C then subsequently terminated by acidification with 2.5% TFA [Trifluoroacetic Acid] to 0.3% final. Digests were desalted using Pierce™ C18 SPE pipette tips (100µl volume) per manufacturer protocol and eluted in 20µl of 70/30/0.1% ACN/H2O/TFA, then dried to completion in the speed-vac and finally reconstituted to 1.25µg/µl final concentration in 0.1% formic acid.

*LC/GC parameters and MS acquisition settings*

Peptides were analyzed by nanoLC-MS/MS using the Agilent 1100 nanoflow system (Agilent) connected to hybrid linear ion trap-orbitrap mass spectrometer (LTQ-Orbitrap Elite™, Thermo Fisher Scientific) equipped with an EASY-Spray™ electrospray source (held at constant 35°C). Chromatography of peptides prior to mass spectral analysis was accomplished using capillary emitter column (PepMap® C18, 3µM, 100Å, 150x0.075mm, Thermo Fisher Scientific) onto which 2µl of extracted peptides was automatically loaded. NanoHPLC system delivered solvents A: 0.1% (v/v) formic acid , and B: 99.9% (v/v) acetonitrile, 0.1% (v/v) formic acid at 0.50 µL/min to load the peptides (over a 30 minute period) and 0.3µl/min to elute peptides directly into the nano-electrospray with gradual gradient from 0% (v/v) B to 30% (v/v) B over 250 minutes followed by rapid gradient from 30% (v/v) B to 50% (v/v) B over 10 minutes and concluded with 7 minute flush-out from 50% (v/v) B to 95% (v/v) B at which time a 2 minute column conditioning at 95% (v/v) B took place. As peptides eluted from the HPLC-column/electrospray source survey MS scans were acquired in the Orbitrap with a resolution of 120,000 followed by CID-type MS/MS fragmentation of 30 most intense peptides detected in the MS1 scan from 350 to 1800 m/z; redundancy was limited by dynamic exclusion.

*Data annotation/validation/analysis procedures*

Elite acquired raw MS/MS data files were converted to mgf file format using MSConvert (ProteoWizard: Open Source Software for Rapid Proteomics Tools Development). Resulting mgf files were used to search against *Neisseria gonorrhoeae* MS11 (2,208 total entries) concurrently with cRAP common lab contaminant database (116 total entries) using in-house Mascot search engine 2.7.0 [Matrix Science] with fixed Cysteine carbamidomethylation and variable Methionine oxidation plus Asparagine or Glutamine deamidation. Peptide mass tolerance was set at 10 ppm and fragment mass at 0.6 Da. Protein annotations, significance of identification and spectral based quantification was done with Scaffold software (version 5.0.1, Proteome Software Inc., Portland, OR). Peptide identifications were accepted if they could be established at greater than 93.0% probability to achieve an FDR less than 1.0% by the Scaffold Local FDR algorithm. Protein identifications were accepted if they could achieve an FDR less than 1.0% and contained at least 2 identified peptides. Protein probabilities were assigned by the Protein Prophet algorithm (1). Proteins that contained similar peptides and could not be differentiated based on MS/MS analysis alone were grouped to satisfy the principles of parsimony. Proteins sharing significant peptide evidence were grouped into clusters. Sequences were analyzed using SignalP 6.0 to predict subcellular localization (2).

## Supplementary Tables and Figures

| Identified Proteins | Molecular Weight | Spectrum Count | SignalP 6.0 |
|---|---|---|---|
| Porin | 38 kDa | 3206 | SPI |
| Chaperonin GroEL | 57 kDa | 1057 | Cyt |
| Peptidoglycan-binding outer membrane protein RmpM | 26 kDa | 441 | SPI |
| LysM peptidoglycan-binding domain-containing protein | 46 kDa | 517 | SPI |
| Outer membrane beta-barrel protein NspA | 18 kDa | 342 | SPI |
| Outer membrane protein assembly factor BamA | 88 kDa | 310 | SPI |
| MliC family protein | 13 kDa | 300 | SPII |
| Autotransporter domain-containing protein | 169 kDa | 263 | SPI |
| Type IV pilus secretin PilQ | 79 kDa | 185 | SPI |
| Outer membrane protein assembly factor BamC | 44 kDa | 168 | SPII |
| Peptidyl-prolyl cis-trans isomerase | 32 kDa | 136 | SPI |
| S41 family peptidase | 53 kDa | 118 | SPI |
| Adhesion and penetration autotransporter App | 160 kDa | 121 | SPI |
| Pyruvate dehydrogenase (acetyl-transferring), homodimeric type | 100 kDa | 124 | Cyt |
| Peptidoglycan DD-metalloendopeptidase family protein | 66 kDa | 99 | SPI |

**Supplementary Table 1.** Proteins in *N. gonorrhoeae* outer membrane vesicle preparations from cell-free supernatant. Fifteen most abundant proteins as determined by total spectral count. Cell-free supernatant was ultracentrifuged at 400,000 x *g* and the pelleted material was digested with try trypsin before mass spectrometry analysis. Sequences were run through SignalP 6.0 to determine subcellular localization. SPI and SPII proteins are transported by the Sec pathway. SPI is cleaved by signal peptidase I, SPII is cleaved by signal peptidase II and predicted to be a lipoprotein. Cyt proteins are not predicted to have signal peptides and thus assumed to be cytoplasmic. Source data are provided in the Source Data file. n = 1

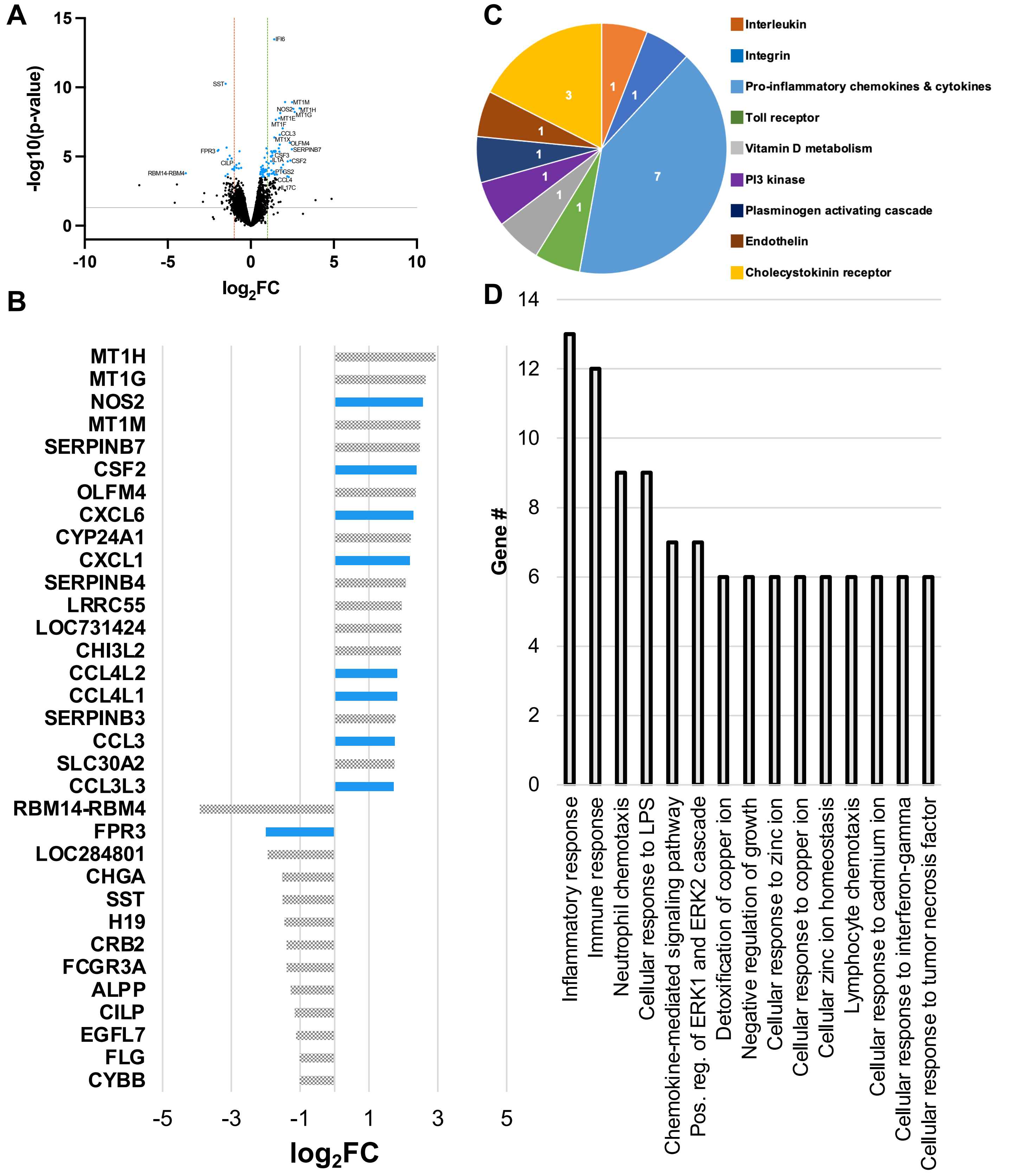


**Supplementary Figure 1.** Transcriptional analysis of 24h *N. gonorrhoeae* supernatant treated human Fallopian tube tissues. **A**) Volcano plot of all genes expressed in mock and 24h *N. gonorrhoeae* supernatant treated tissues. Each dot represents one gene. Those colored black represent genes with a false discovery rate (FDR) > 0.05 while blue dots represent those with an FDR < 0.05. Dots below the gray line represent genes with $p > 0.05$ (-log10(p-value) < 1.3) while those above the line represent those with $p < 0.05$. Genes to the left of the red line are repressed 2-fold or greater ($log_2FC \geq 1$). Genes to the right of the green line are induced 2-fold or greater. "FC" = fold change. **B**) Relative expression levels ($log_2FC$) of genes significantly different (FDR < 0.05, $p < 0.05$, $log_2FC \geq 1$) between treatments. All significantly repressed genes ($log_2FC \leq -1$) are displayed. Only the top 20 significantly induced genes ($log_2FC \geq 1$) are displayed. Blue bar color is indicative of assignment to a PANTHER, GO, or KEGG inflammation pathway. For A and B, the posterior probability of differential expression and the FDR estimate for multiple comparisons were determined by a Bayesian model (*EBSeq*). **C**) PANTHER pathway analysis. The number within each slice is the number of significantly different genes assigned to the pathway. **D**) Gene ontology (GO) biological function pathway analysis, top 15 pathways. Source data are provided in the Source Data file.

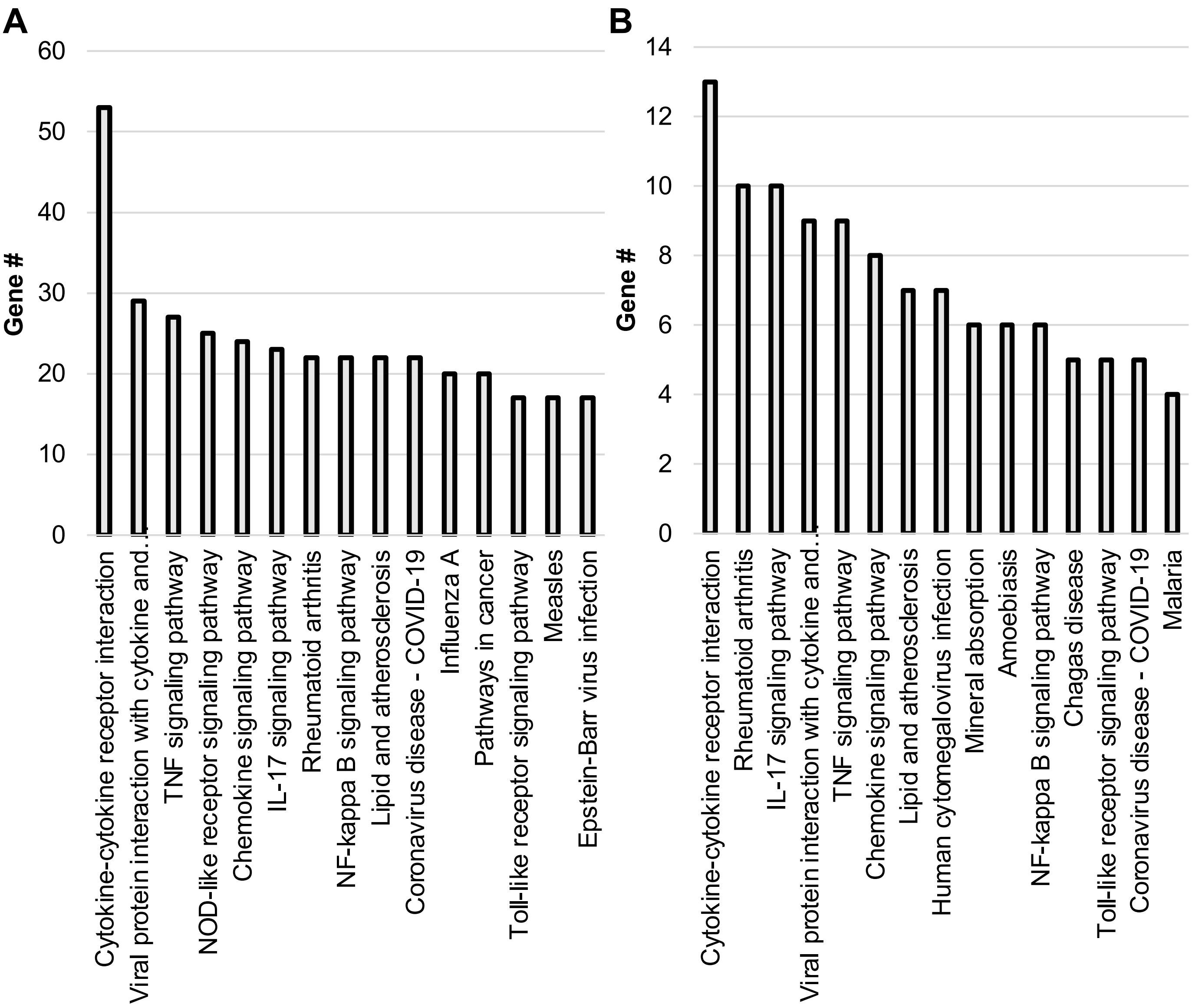


**Supplementary Figure 2**. KEGG pathway analysis. **A**) The top 15 pathways significantly associated with genes upregulated upon 6h treatment with wild type *N. gonorrhoeae* supernatant. **B**) The top 15 pathways significantly associated with genes upregulated upon 24h treatment with wild type *N. gonorrhoeae* supernatant. Source data are provided in the Source Data file.

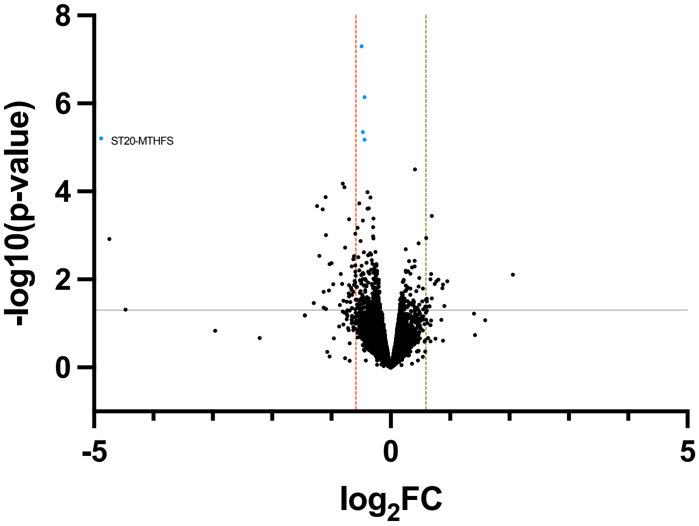


**Supplementary Figure 3**. Transcriptional analysis of 24h *ΔldcA* treated human Fallopian tube tissues. Volcano plot of all genes expressed in 24h wild type and *ΔldcA* treated tissues. Each dot represents one gene. Those colored black represent genes with a false discovery rate (FDR) > 0.05 while blue dots represent those with an FDR < 0.05. Dots below the gray line represent genes with p > 0.05 (-log10(p-value) < 1.3) while those above the line represent those with p < 0.05. Genes to the left of the red line are repressed 1.5-fold or greater ($log_2FC \geq 0.59$). Genes to the right of the green line are induced 1.5-fold or greater. "FC" = fold change. the posterior probability of differential expression and the FDR estimate for multiple comparisons were determined by a Bayesian model (*EBSeq*). Source data are provided in the Source Data file.

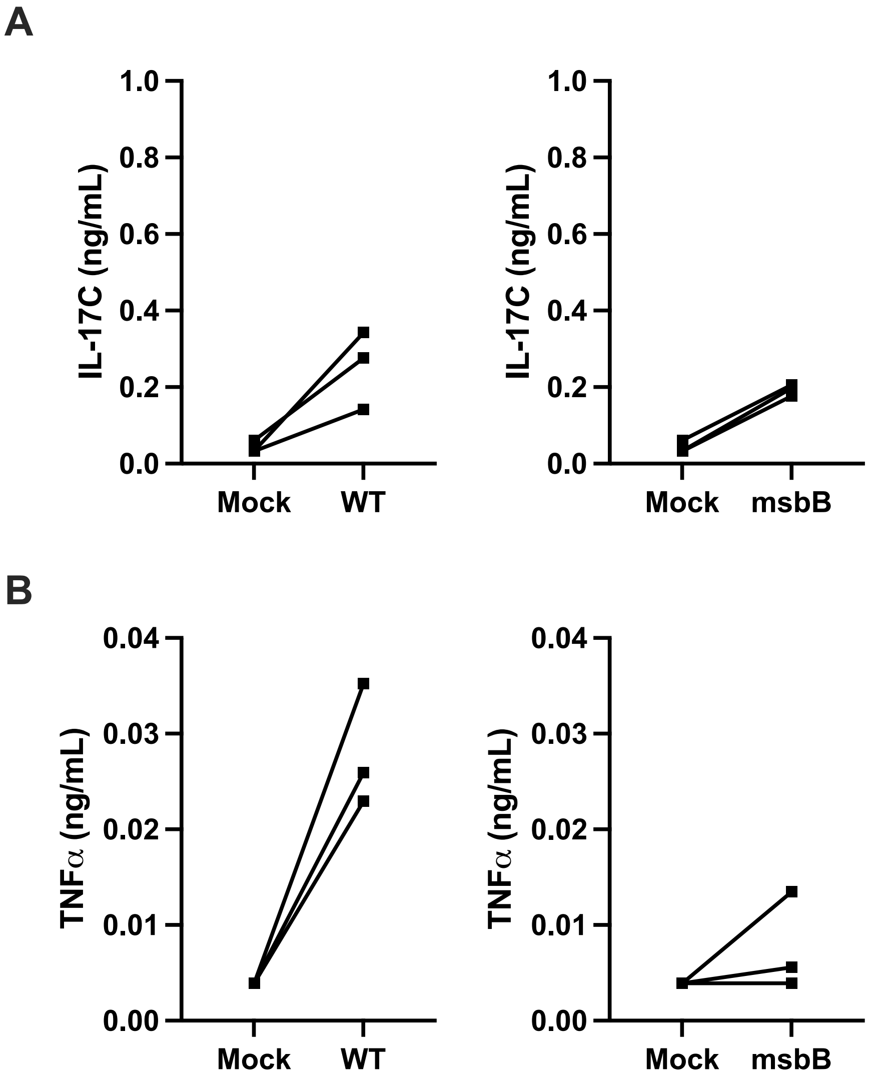


**Supplementary Figure 4**. ELISA quantification of wild type and *msbB* mutant supernatant treated FTOC. **A**) Comparison of IL-17C production in 24h mock and supernatant treated Fallopian tube tissues. **B**) Comparison of TNF-α production in 24h mock and supernatant treated Fallopian tube tissues. n=3 biologically independent samples. Lines between points represent donor pairs. Each line represents an independent experiment in different donors. WT = wild type strain MS11, msbB = lipooligosaccharide mutant in the MS11 background. Source data and exact p-values are provided in the Source Data file. A two-tailed, paired t-test was used for comparisons.

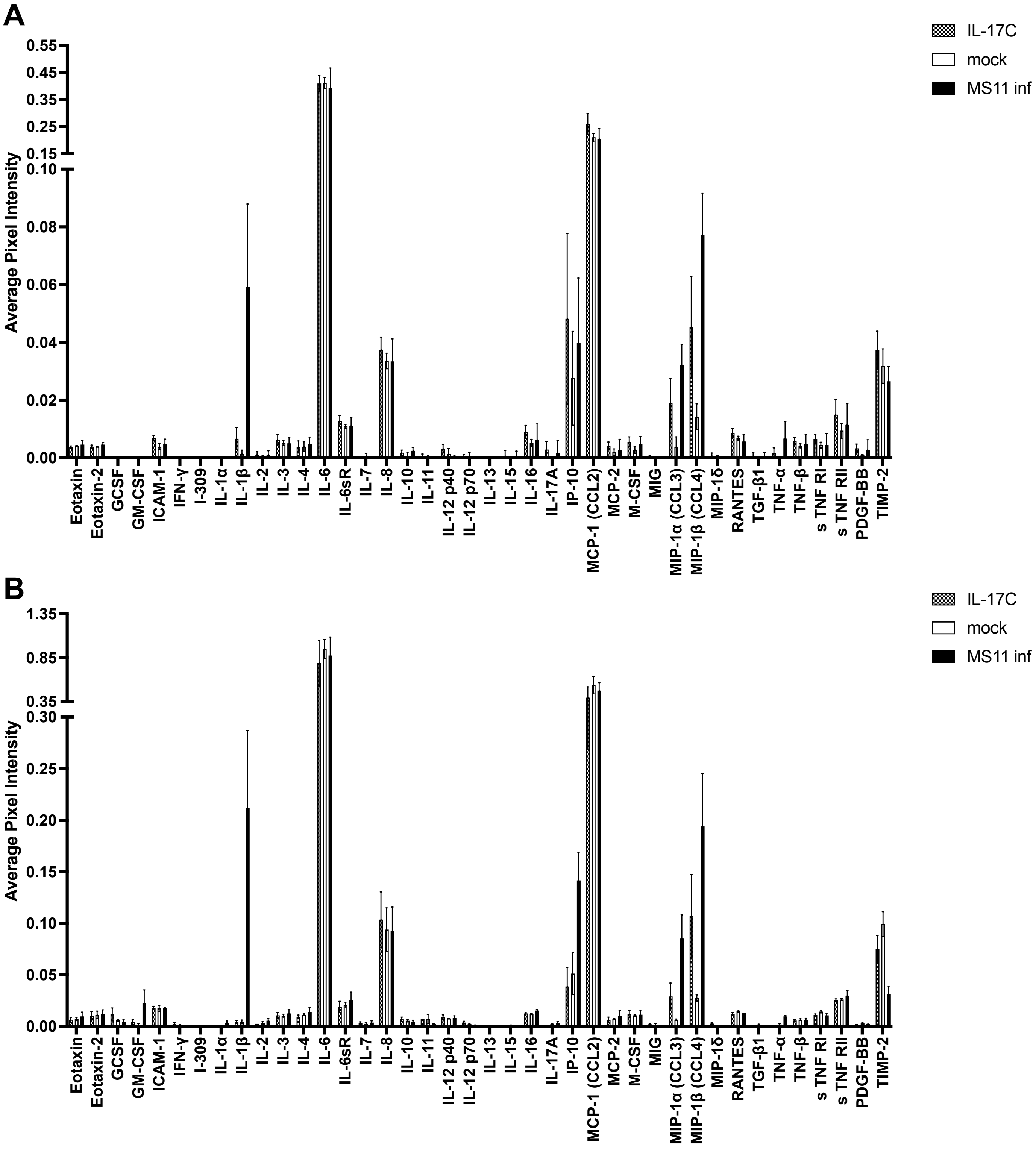


**Supplementary Figure 5**. Cytokine array of IL-17C or *N. gonorrhoeae* treated FTOC. **A**) Cytokine response of FTOC treated with 200ng rhIL-17C or live gonococci for 6h, n=3 biologically independent samples **B**) Cytokine response of FTOC treated with 200ng rhIL-17C or live gonococci for 24h, n=2 biologically independent samples. Error bars represent SEM. Source data are provided in the Source Data file.

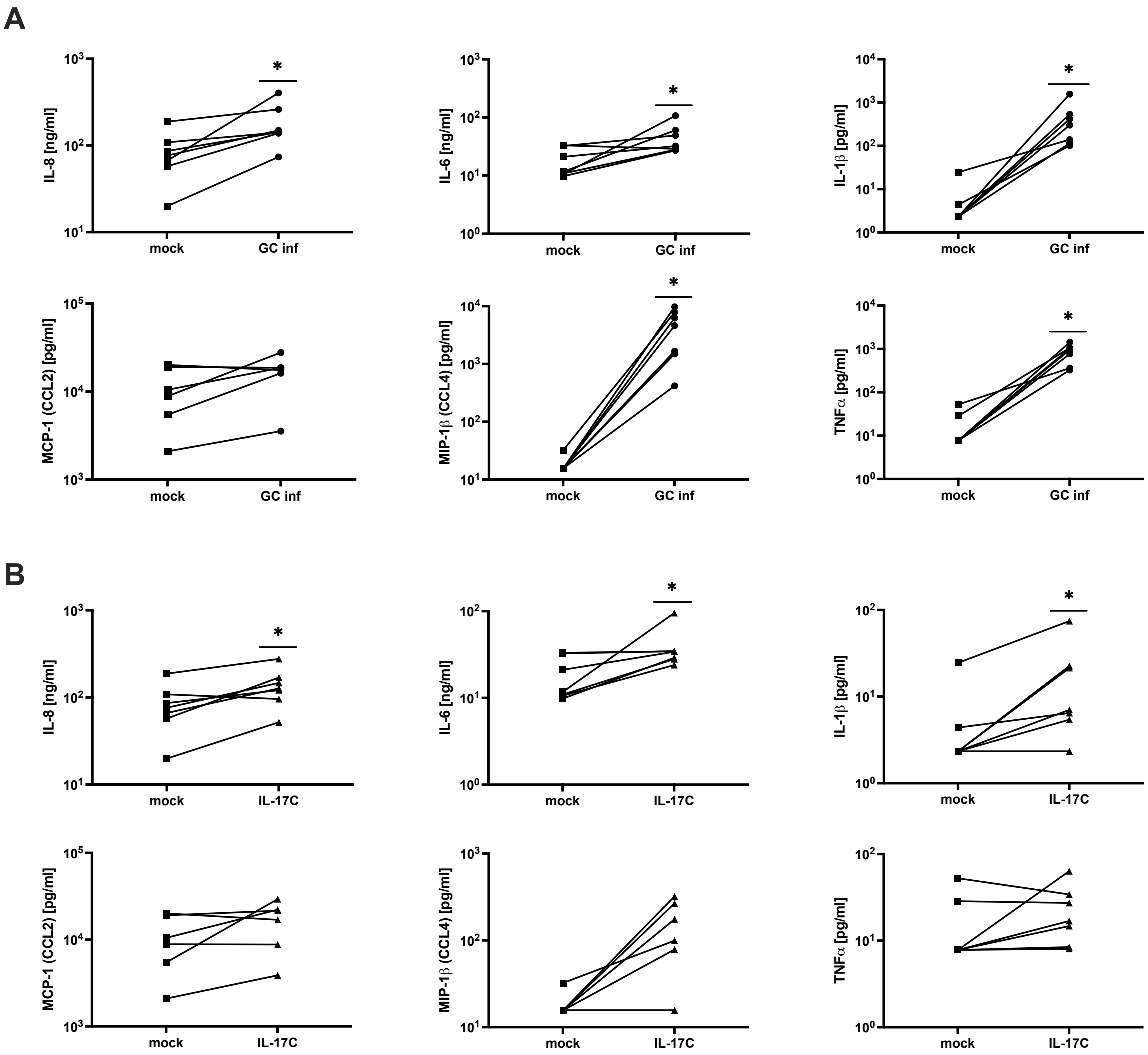


**Supplementary Figure 6**. ELISA quantification of 24h IL-17C and *N. gonorrhoeae* treated FTOC. **A**) Comparison of 24h mock (square) and *N. gonorrhoeae* infected (circle) Fallopian tube tissues. n=7 biologically independent samples, except in CCL2 where n=6 **B**) Comparison of mock (square) and 24h 200ng rhIL-17C (triangle) treated Fallopian tube tissues. n=7 biologically independent samples, except in CCL2 where n=6 . Lines between points represent donor pairs. Each line represents an independent experiment in different donors. * $p < 0.05$ Source data and exact p-values are provided in the Source Data file.

## Supplementary References


1. Nesvizhskii AI, Keller A, Kolker E, Aebersold R. 2003. A statistical model for identifying proteins by tandem mass spectrometry. Anal Chem. 75(17):4646-4658.
2. Teufel F, Almagro Armenteros JJ, Johansen AR, Gíslason MH, Pihl SI, Tsirigos KD, Winther O, Brunak S, von Heijne G, Nielsen H. 2022. SignalP 6.0 predicts all five types of signal peptides using protein language models. Nature Biotechnology. 40(7):1023–1025.
