# Supplementary material for: IL-17C is a driver of damaging inflammation during Neisseria gonorrhoeae infection of human Fallopian tube
Source: Nat Commun. 2024 May 4;15:3756. doi: 10.1038/s41467-024-48141-3 (PMC11069574; doi:10.1038/s41467-024-48141-3)
Supplement: Supplementary file 3 — Description of Additional Supplementary Files [file 41467_2024_48141_MOESM3_ESM.pdf]

Description of Additional Supplementary Files

File Name: Data S1
Description: Pathway analysis of WT vs *ΔldcA* mutant treated tissues at 24h. Gene ontology (GO) biological function pathway analysis.
