# Supplementary material for: IL-17C is a driver of damaging inflammation during Neisseria gonorrhoeae infection of human Fallopian tube
Source: Nat Commun. 2024 May 4;15:3756. doi: 10.1038/s41467-024-48141-3 (PMC11069574; doi:10.1038/s41467-024-48141-3)
Supplement: Supplementary file 5 — Reporting Summary [file 41467_2024_48141_MOESM5_ESM.pdf]

nature portfolio

Corresponding author(s): Joseph Dillard

Last updated by author(s): 4/6/2024

Reporting Summary

Nature Portfolio wishes to improve the reproducibility of the work that we publish. This form provides structure for consistency and transparency in reporting. For further information on Nature Portfolio policies, see our Editorial Policies and the Editorial Policy Checklist.

Statistics

For all statistical analyses, confirm that the following items are present in the figure legend, table legend, main text, or Methods section.

n/a	Confirmed	
☐	☒	The exact sample size (*n*) for each experimental group/condition, given as a discrete number and unit of measurement
☐	☒	A statement on whether measurements were taken from distinct samples or whether the same sample was measured repeatedly
☐	☒	The statistical test(s) used AND whether they are one- or two-sided<br>*Only common tests should be described solely by name; describe more complex techniques in the Methods section.*
☒	☐	A description of all covariates tested
☒	☐	A description of any assumptions or corrections, such as tests of normality and adjustment for multiple comparisons
☐	☒	A full description of the statistical parameters including central tendency (e.g. means) or other basic estimates (e.g. regression coefficient) AND variation (e.g. standard deviation) or associated estimates of uncertainty (e.g. confidence intervals)
☐	☒	For null hypothesis testing, the test statistic (e.g. *F*, *t*, *r*) with confidence intervals, effect sizes, degrees of freedom and *P* value noted<br>*Give P values as exact values whenever suitable.*
☒	☐	For Bayesian analysis, information on the choice of priors and Markov chain Monte Carlo settings
☒	☐	For hierarchical and complex designs, identification of the appropriate level for tests and full reporting of outcomes
☒	☐	Estimates of effect sizes (e.g. Cohen's *d*, Pearson's *r*), indicating how they were calculated

Our web collection on statistics for biologists contains articles on many of the points above.

Software and code

Policy information about availability of computer code

Data collection	Nikon NIS Elements software was used for the collection of IHC images in Fig. 4.
Data analysis	Skewer<br>STAR (v2.4.0j)<br>RSEM (v1.2.16)<br>EBSeq (v1.1.5)<br>LiCor Image Studio<br>Adobe Photoshop CS5<br>GraphPad Prism (v4.0)<br>MSConvert<br>Scaffold software (v5.0.1)<br>Protein Prophet algorithm<br>SignalP (v6.0)

For manuscripts utilizing custom algorithms or software that are central to the research but not yet described in published literature, software must be made available to editors and reviewers. We strongly encourage code deposition in a community repository (e.g. GitHub). See the Nature Portfolio guidelines for submitting code & software for further information.

Data

Policy information about availability of data

All manuscripts must include a data availability statement. This statement should provide the following information, where applicable:

- Accession codes, unique identifiers, or web links for publicly available datasets
- A description of any restrictions on data availability
- For clinical datasets or third party data, please ensure that the statement adheres to our policy

RNA sequencing data supporting this study's findings have been deposited in the Gene Expression Omnibus (GEO) database under the accession identifier GSE253499 [https://www.ncbi.nlm.nih.gov/geo/query/acc.cgi?acc=GSE253499]. The uploaded data include raw counts as fastq files for each sample and processed counts as transcripts per million (TPM). The mass spectrometry data generated in this study have been deposited to the ProteomeXchange Consortium database via the PRIDE partner repository under accession code PXD051157 [http://www.ebi.ac.uk/pride/archive/projects/PXD051157]. The following databases were used for data analysis: DAVID Bioinformatics Database, version 2021 [https://david.ncifcrf.gov/home.jsp]; PANTHER classification system, version 17.0 [http://www.pantherdb.org]; Mascot search engine, in-house v2.7.0. Source data are provided with this paper.

Research involving human participants, their data, or biological material

Policy information about studies with human participants or human data. See also policy information about sex, gender (identity/presentation), and sexual orientation and race, ethnicity and racism.

Reporting on sex and gender	Our findings only apply to the human female sex, as Fallopian tubes are not present in the human male sex.
Reporting on race, ethnicity, or other socially relevant groupings	No categorization variables were used.
Population characteristics	Participants were aged 50 years or younger, never had received radiation or chemotherapy, and were seeking care for a hysterectomy and/or salpingectomy.
Recruitment	Participants were recruited by a 3rd party entity, from which de-identified tissue was purchased, based on the criteria above.
Ethics oversight	Use of de-identified human tissue for these studies was approved by the Institutional Review Board of the University of Wisconsin-Madison, Protocol #2014-0874, and determined to be exempt as human subjects research.

Note that full information on the approval of the study protocol must also be provided in the manuscript.

Field-specific reporting

Please select the one below that is the best fit for your research. If you are not sure, read the appropriate sections before making your selection.

☒ Life sciences ☐ Behavioural & social sciences ☐ Ecological, evolutionary & environmental sciences

For a reference copy of the document with all sections, see nature.com/documents/nr-reporting-summary-flat.pdf

Life sciences study design

All studies must disclose on these points even when the disclosure is negative.

Sample size	Samples sizes were between 3-7. No sample size calculation was performed. Sample sizes were determined by primary tissue availability, quality, and size.
Data exclusions	For all cytokine ELISAs, a 20% standard deviation cutoff was used for inclusion.
Replication	At least 3 independent replicates were performed for every tissue experiment*, with each replication using tissue from a different donor.*Apart from the data presented in figure S5B, which has only 2 replicates. Mass spec data in table S1 are from a single replicate. All attempts at replication were successful.
Randomization	NA We performed in vitro experiments and were able to use treatments or no treatment controls on tissue samples from the same individuals.
Blinding	NA We performed in vitro experiments on tissue from donors and used treatment and no treatment controls on tissue samples from the same individuals. Quantitative measures such as RNA-Seq and ELISAs were used for assessments and are not subjective. Microscopy was used for certain plus or minus determinations and included controls for comparison that are shown in the figures.

Reporting for specific materials, systems and methods

We require information from authors about some types of materials, experimental systems and methods used in many studies. Here, indicate whether each material, system or method listed is relevant to your study. If you are not sure if a list item applies to your research, read the appropriate section before selecting a response.

Materials & experimental systems

n/a	Involved in the study	
☐	☒	Antibodies
☒	☐	Eukaryotic cell lines
☒	☐	Palaeontology and archaeology
☒	☐	Animals and other organisms
☒	☐	Clinical data
☒	☐	Dual use research of concern
☒	☐	Plants

Methods

n/a	Involved in the study	
☒	☐	ChIP-seq
☒	☐	Flow cytometry
☒	☐	MRI-based neuroimaging

Antibodies

Antibodies used: Primary anti IL-17RE: NBP1-93925, Novus Bio; Secondary: MP-7401, Vector Laboratories

Validation: According to the manufacturer's website, the specificity of the primary antibody (NBP1-93925) was verified on a Protein Array containing target protein plus 383 other non-specific proteins. Images are provided of successful use for immunohistochemistry, including on Fallopian tube. https://www.novusbio.com/products/il-17re-antibody_nbp1-93925#datasheet

Plants

Seed stocks: *Report on the source of all seed stocks or other plant material used. If applicable, state the seed stock centre and catalogue number. If plant specimens were collected from the field, describe the collection location, date and sampling procedures.*

Novel plant genotypes: *Describe the methods by which all novel plant genotypes were produced. This includes those generated by transgenic approaches, gene editing, chemical/radiation-based mutagenesis and hybridization. For transgenic lines, describe the transformation method, the number of independent lines analyzed and the generation upon which experiments were performed. For gene-edited lines, describe the editor used, the endogenous sequence targeted for editing, the targeting guide RNA sequence (if applicable) and how the editor was applied.*

Authentication: *Describe any authentication procedures for each seed stock used or novel genotype generated. Describe any experiments used to assess the effect of a mutation and, where applicable, how potential secondary effects (e.g. second site T-DNA insertions, mosiacism, off-target gene editing) were examined.*
